# Molybdenum Carbide and Sulfide Nanoparticles as Selective Hydrotreating Catalysts for FCC Slurry Oil to Remove Olefins and Sulfur

**DOI:** 10.3390/nano11102721

**Published:** 2021-10-15

**Authors:** He Liu, Zhipeng Qiu, Huihui Pan, Aijun Guo, Shouhui Jiao, Feng Wang, Kun Chen, Zongxian Wang

**Affiliations:** State Key Laboratory of Heavy Oil Processing, College of Chemical Engineering, China University of Petroleum (East China), 66 Changjiang West Road, Huangdao District, Qingdao 266580, China; liuhe@upc.edu.cn (H.L.); panhuihui_upc@163.com (H.P.); b18030013@s.upc.edu.cn (S.J.); wfzgsydx123@163.com (F.W.); chenkun@upc.edu.cn (K.C.); james_w3112@163.com (Z.W.)

**Keywords:** molybdenum carbide nanoparticles, molybdenum sulfide nanoparticles, selective hydrotreating, FCC slurry oil, olefins and sulfur removal

## Abstract

As the two types of major impurities in FCC slurry oil (SLO), olefins and sulfur seriously deteriorate the preparation and quality of mesophase pitch or needle coke. The development of a hydrotreatment for SLO to remove olefins and sulfur selectively becomes imperative. This work presents the potentiality of dispersed Mo_2_C and MoS_2_ nanoparticles as selective hydrotreating catalysts of SLO. Mo_2_C was synthesized by the carbonization of citric acid, ammonium molybdate and KCl mixtures while MoS_2_ was prepared from the decomposition of precursors. These catalysts were characterized by XRD, HRTEM, XPS, BJH, BET, and applied to the hydrotreating of an SLO surrogate with defined components and real SLO. The conversion of olefins, dibenzothiophene and anthracene in the surrogate was detected by GC-MS. Elemental analysis, bromine number, diene value, ^1^H-NMR and spot test were used to characterize the changes of the real SLO. The results show that hydrotreating the SLO surrogate with a very small amount of Mo-based nanoparticles could selectively remove olefins and sulfur without the overhydrogenation of polyaromatics. Mo_2_C exhibited much better activity than MoS_2_, with 95% of olefins and dibenzothiophene in the surrogate removed while only 15% anthracene was hydrogenated. The stability of the real SLO was significantly improved. Its structural parameters changed subtly, proving the aromatic macromolecules had been preserved.

## 1. Introduction

Slurry oil (SLO) is an important byproduct in the fluidized catalytic cracking (FCC) process. Due to the ever-increasing supply of heavy oil with the short fall of conventional crudes and persistent high demand for light fuels, the production of SLO rises and its quality inevitably becomes inferior [1]. Since SLO is enriched with 3–5 rings of polycyclic aromatic hydrocarbons (PAHs) with short side chains, it is widely known as an excellent potential raw material to produce mesophase pitch and needle coke, which could heighten its utilization value remarkably [2,3,4]. Nevertheless, the impurities in SLO could seriously deteriorate the quality of the prepared carbonaceous material, among which sulfur and olefins are the most critical factors [5,6,7]. The sulfur in needle coke with a content higher than 0.5 wt% can cause irreversible volume expansion (i.e., puffing) during graphitization heat treatment, reducing the strength and electrical conductivity of the electrodes [8,9]. In the meantime, the olefins are important chemically active intermediates and products in the FCC process. Our group has identified the olefins widely distributed in SLO, and found that they could worsen the thermal stability of SLO, induce a premature coke of SLO thermal processing, and hamper the orderly development of mesophase pitch [7,10]. Therefore, as the most practical and efficient way to remove olefins and sulfur, the selective hydrotreating of SLO is imperative for chemical structure modification of SLO and deserves great attention for high-value SLO utilization.

The hydrotreating of various straight-run or cracking distillates (i.e., gasoline, diesel, and vacuum gas oil) and residues is extensively achieved using the transition metal sulfides supported on porous materials as catalysts [11,12,13,14,15]. Despite the pioneering research of SLO hydrotreating over alumina-supported Co-Mo or Ni-Mo catalysts that has been reported, their main concern was to remove the sulfur in SLO and thus control the sulfur levels in needle coke [16,17,18,19]. In contrast, detailed information on olefins removal of SLO through selective hydrotreating is extremely limited. As stated by Abrahamson et al. [18], the aromatic/aliphatic hydrocarbon ratio is an important structural parameter of SLO for coke morphology modification. Hence, an efficient catalyst for the selective hydrotreating of SLO should possess a high activity for sulfur and olefins removal, but a low activity for the hydrogenation of PAHs.

To date, molybdenum-based nanoparticles (e.g., MoS_2_ and Mo_2_C) have attracted great attention for hydrogenation reactions because of their high abundance and low cost [20,21,22]. MoS_2_ is well known as the catalytic active sites for dispersed catalyst in the slurry-phase hydrocracking process of heavy oil [23,24]. It can be prepared in situ from water-soluble and oil-soluble catalytic precursors and thus dispersed in the oil system, which could avoid the block issue of the catalyst bed. Meanwhile, the relevant researchers suggested that MoS_2_ could simultaneously display catalytic activity for aromatics hydrogenation during hydrotreating, resulting in a reduced selectivity for sulfur and olefins removal [25]. The ability of dispersed MoS_2_ for the transformation of bicyclic aromatics was discussed by Deng et al. in detail [26]. The hydrotreatment of light cycle oil over a dispersed MoS_2_ catalyst, as conducted by Zhang et al. [25], found that about 81% of the bicyclic aromatics were hydrogenated to monocyclic aromatics while the monocyclic aromatics and polyaromatics were barely eliminated. Conversely, the exploration of Hu et al. indicated that phenanthrene could be converted to different hydrogenated intermediates on a dispersed MoS_2_ catalyst [27]. The hydrogenation of naphthalene, phenanthrene, and pyrene were observed over MoS_2_ by Dutta et al. [28], and the conversion was higher than 50%. Clearly, the hydrogenation selectivity of MoS_2_ for olefins and sulfur compounds remains to be revealed in the case of the overhydrogenation of aromatics. Kaluža et al. [14] even found that the MoS_2_ catalysts exhibited higher selectivity to the hydrogenation of olefins (HYDO) while the CoMoS and NiMoS showed lower selectivity towards HYDO during the hydrogenation of 1-benzothiophene/1-methyl-1-cyclohexene. It further suggests MoS_2_ might be a better candidate for the deep hydrogenation of olefins. In addition to MoS_2_, molybdenum carbide (Mo_2_C) nanoparticles showed excellent catalytic performance for electrocatalytic hydrogen evolution, water-gas shift reaction, hydrodesulfurization, hydrodeoxygenation and hydrodenitrogenation because of the platinum (Pt)-like d-band electronic structure by inducing carbon into the metal lattice [29,30,31,32,33,34,35,36,37,38]. Compared with noble metal catalysts, Mo_2_C catalysts are of higher sulfur tolerance and better stability. As reported by Aegerter et al. [39], β-Mo_2_C possessed a higher thiophene HDS activity than MoS_2_, and had the potential to replace MoS_2_ in commercial HDS reactors. Sajkowski et al. [40] studied the hydrotreating of coal-derived gas oil and residuum over Mo_2_C/Al_2_O_3_, and the results showed that the catalytic rates over Mo_2_C could be as much as five times that of MoS_2_/A1_2_O_3_. Qiu et al. [41] conducted the hydrodenitrogenation of quinoline over β-Mo_2_C. It was found that Mo_2_C exhibited remarkable selectivity for denitrogenation and low activity for aromatic ring destruction. According to the available literature, the Mo_2_C catalysts have never been utilized in the selective hydrotreating of SLO.

In this paper, Mo_2_C was synthesized ex situ by the carbonization of citric acid, ammonium molybdate and KCl mixtures while MoS_2_ was obtained in situ from the decomposition of water-soluble and oil-soluble precursors. These catalysts were characterized in detail by X-ray diffraction (XRD), high resolution transmission electron microscopy (HRTEM), X-ray photoelectron spectroscopy (XPS), Brunauer Emmett Teller (BET) and Barrett-Joyner-Halenda analysis (BJH), and then applied for the hydrotreating of the SLO surrogate with defined components and the real SLO. The conversion of different model compounds in the surrogate was detected by GC-MS. Elemental analysis, bromine number, diene value, ^1^H-NMR and spot test were used to characterize the changes of the real SLO. The potentiality of Mo_2_C and MoS_2_ nanoparticles as selective hydrotreating catalysts of SLO to remove olefins and sulfur was discussed.

## 2. Materials and Methods

### 2.1. Preparation of Catalysts

The purity and suppliers of the chemicals used in the present work are provided in Table S1 as supporting information. A total of 4.0 g ammonium molybdate tetrahydrate (AMT) and 1.2451 g citric acid monohydrate (CAM), 0.338 g potassium chloride and 0.8044 g ethylene glycol were mixed with 50 mL deionized water, and the pH value of the solution was kept at 1.5 by adding nitric acid. The wet gel was prepared by removing water with a rotary evaporator, and then vacuum dried at 110 °C for 4.0 h. The resultant powder was ground to 60–100 mesh and carbonized under nitrogen with a flow rate of 100 L/min at 800 °C with a heating rate of 5 °C/min. After the carbonization, the sample was passivated at 800 °C for 2 h using 1% O_2_-Ar mixture to obtain the final Mo_2_C catalyst.

The MoS_2_ catalysts were prepared by solvothermal method from oil-soluble precursor molybdenum dialkyl dithiocarbamate (Mo-DTC) and water-soluble precursor ammonium molybdate tetrahydrate (AMT), which were designated as O-MoS_2_ and W-MoS_2_, respectively. Mo-DTC was decomposed and self-sulfurized in 1-methylnaphthalene media under hydrogen at the optimized temperature of 380 °C for 45 min to obtain O-MoS_2_ catalyst. For preparation of W-MoS_2_, AMT was decomposed and sulfurized using sulfur as the sulfiding agent at the optimized temperature of 340 °C for 30 min. To prevent the oxidation as much as possible, MoS_2_ samples were soaked in ethanol before characterization.

### 2.2. Characterization of Catalysts

The crystal structure of the samples was characterized by using XRD system (X‘Pert Pro MPD) equipped with a Cu-Kα irradiation source in the 2*θ* range of 5–75° with a scanning speed of 5°·min^−1^. The morphologies of samples were measured by TEM (JEM-2100UHR, Japan Electronics Co., Ltd., Tokyo, Japan) at 5 kV accelerated voltage. XPS analysis was performed on a K-alpha 250Xi spectrometer (Thermo Fisher Scientific, Waltham, MA, USA) with an Ar ion source at 0.5–1.5 KeV. BET specific surface areas and BJH pore size distribution of catalysts were determined based on the nitrogen physisorption measurement using ASAP 2020M/Micromeritics instrument (Micromeritics Instrument Co., Norcross, GA, USA). During nitrogen physisorption measurement, the wet MoS_2_ samples with ethanol were degassed at 150 °C under vacuum for 10 h before analysis.

### 2.3. Hydrotreating of SLO Surrogate and SLO

Based on our previous work [7], the readily available conjugated olefins and monoolefin (styrene, trans-stilbene, and 1-octene) were used as the olefin model compounds due to their similar structures to the native olefins identified in slurry oil. Dibenzothiophene and anthracene were selected as model of the sulfur-containing and aromatic compound, respectively. The bulk solvent of simulated slurry oil was determined as 1-methylnaphthalene. The weight percentages of the five components in 1-methylnaphthalene are 3.33% styrene, 3.33% trans-stilbene, 3.33% 1-octene, 1% dibenzothiophene and 1.5% anthracene. Main properties of the real SLO feedstock are listed in Table 1.

The hydrotreating experiments were carried out in a 500 mL autoclave reactor. The 100 g SLO surrogate or 200 g SLO feed and the certain amounts of Mo_2_C or Mo-DTC (calculated as 50 ug·g^−1^ Mo metal content) were mixed under ultrasonic for about 30 min to ensure good dispersion and transferred into the reactor. The added amount of Mo-AMT was determined based on 100 ug·g^−1^ Mo metal content because of its commonly low catalytic activity due to poor dispersion. A measure of 1 wt% sorbitan monooleate (Span-80) was added as the emulsifier to enhance the dispersion. Mo_2_C particles with 60–100 mesh were prepared ex situ and preactivated under hydrogen to remove the surface passivation layer. Even though the internal diffusion limitations cannot be eliminated completely, it was believed that they can be low by taking these dispersion measures. After purging with nitrogen three times to remove air, the autoclave was pressurized with 4.0 MPa of H_2_. For the reaction systems with Mo precursors, it was first subjected to the presulfiding treatment similar to the description in Section 2.1. Subsequently, the reactor was heated to 380 °C within 30 min and maintained for 2 h. The time when temperature reached about 378 °C was taken as zero. The reaction systems were stirred at 800 r/min to eliminate the external diffusion limitation as much as possible. After that, it was quenched in cooling water to obtain the final products.

### 2.4. Analysis of the Products

The reaction products of SLO surrogate hydrotreating were detected by gas chromatography (GC, 450-GC, Bruker Daltonics, Billerica, MA, USA) and gas chromatography-mass (GC-MS, QP201, Shimadzu, Kyoto, Japan) spectrometry. The olefin distribution of slurry oil after hydrotreating was measured by bromine index analyzer (JF-3, RISHANG Instrument Manufacturing Co., Ltd, Daqing, China). Conjugated olefin distribution of slurry oil was determined by diene value based on the ASTM UOP326-2008. The element content of slurry oil was detected by an elemental analyzer (Vario EL III, Elementar, Hanau, Germany). The changes in hydrogen distribution of slurry oil were analyzed by the ^1^H-NMR spectrum. It was completed on the av500/BRUKER ^1^H-NMR spectrometer using deuterated chloroform as solvent and tetramethylsilane as internal standard, and the NMR frequency was 500 MHz. The stability of SLO was evaluated by spot experiment according to ASTM-D4740-02 standard.

## 3. Results and Discussion

### 3.1. Catalysts Characterization

**Figure 1:**
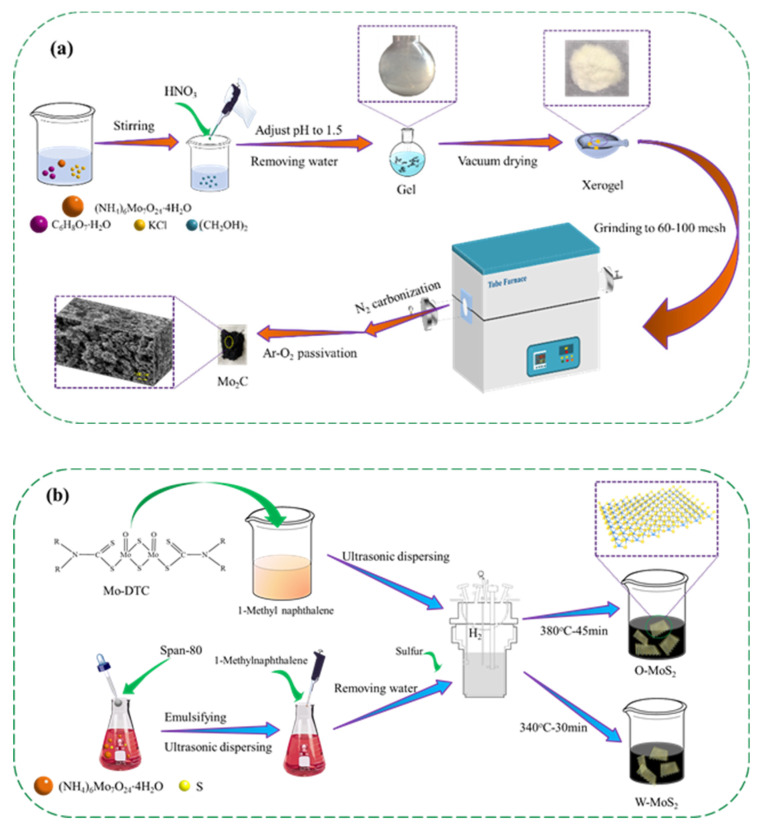
Schematic illustration of the preparation process of the catalysts, (**a**) Mo_2_C, and (**b**) O-MoS_2_ and W-MoS_2_.

To explore the morphology of the Mo-based nanoparticles, Mo_2_C and MoS_2_ were prepared according to the steps illustrated in Figure 1 and characterized in detail.

**Figure 2:**
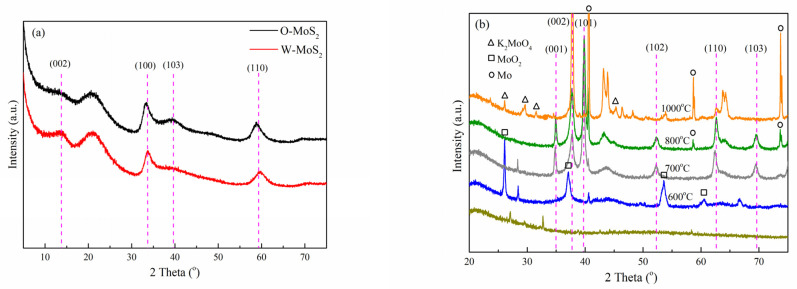
XRD patterns of catalysts, (**a**) MoS_2_, and (**b**) Mo_2_C.

XRD measurement was employed to analyze the crystalline structure of the catalysts, with the patterns displayed in Figure 2. Based on the PDF#74-0932 card, the identified diffraction peaks for O-MoS_2_ and W-MoS_2_ can be indexed to the hexagonal 2H-MoS_2_ phase [42]. The weak intensity of peaks at 32.7° and 58.3°, corresponding to the (100) and (110) in-plane diffraction, indicates poor crystallinity and the small crystalline domains of MoS_2_. Furthermore, the (002) peak representative of the number of stacked layers was weaker in O-MoS_2_ than that in W-MoS_2_, indicating that as-synthesized O-MoS_2_ showed no evident stacking of the MoS_2_ monolayers along the c-axis while W-MoS_2_ could be of a multiplayer nature. Based on the Scherrer equation, the mean sizes of W-MoS_2_ and O-MoS_2_ were tentatively calculated to be 4.1 and 4.4 nm, respectively.

For the XRD pattern of Mo_2_C nanoparticles obtained at different carbonization temperatures, it can be seen that the carbonization was incomplete under 600 °C since the characteristic peaks at 25.95°, 36.94°, 53.44° and 60.19° corresponding to monoclinic molybdenum dioxide (MoO_2_) were obviously observed. The (001), (002), (101), (102), (110), (103), and (200) peaks of β-Mo_2_C, which has a hexagonal structure and high thermal stability, began to appear at 700 °C [43]. When the carbonization temperature was increased to 1000 °C, the characteristic diffraction peaks corresponding to K_2_MoO_4_ (2*θ* = 26.09°, 29.65°, 31.53° and 45.35°) and metallic molybdenum (2*θ* = 40.49°, 58.61° and 73.66°) were clearly visible. Meanwhile, the high temperature could easily lead to the sintering of the active phase, which could destroy its structure and affect the catalytic activity. In this study, Mo_2_C nanoparticles prepared at a carbonization temperature of 800 °C were selected as the catalyst in the follow-up study to ensure sufficient Mo_2_C formation and fewer byproducts. The mean size of Mo_2_C nanoparticles was estimated to be 22.6 nm, approximately.

**Figure 3:**
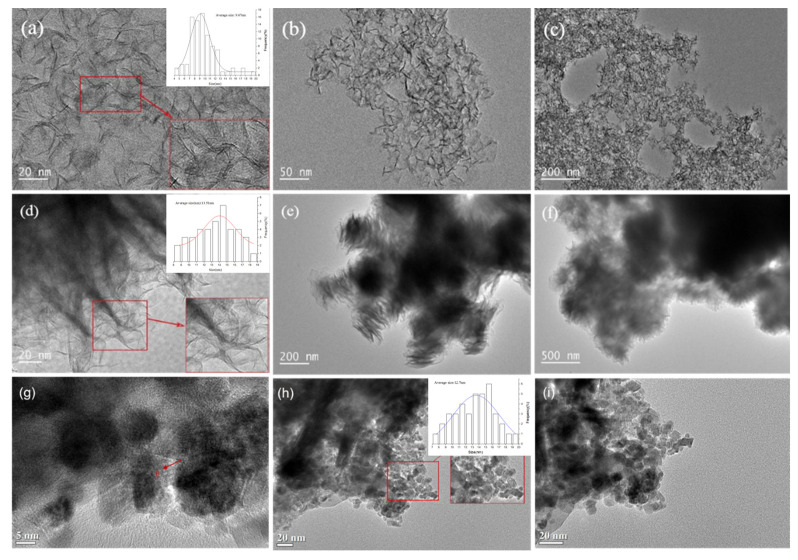
HRTEM pictures of catalysts, (**a**–**c**) O-MoS_2_, (**d**–**f**) W-MoS_2_, (**g**–**i**) Mo_2_C.

**Figure 4:**
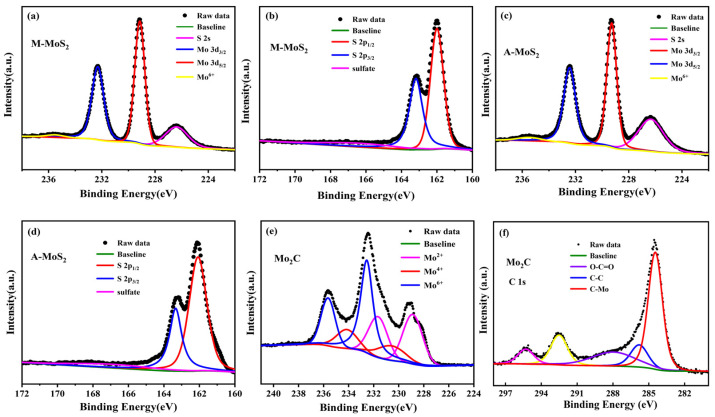
High-resolution XPS spectra of the Mo 3d (**a**,**c**), S 2p (**b**,**d**) regions of O-MoS_2_ and W-MoS_2_ respectively, and the Mo 3d (**e**), C 1s (**f**) regions of Mo_2_C.

Figure 3 displays the HRTEM images of O-MoS_2_, W-MoS_2_ and Mo_2_C catalysts. MoS_2_ presented a uniformly distributed sheet structure with a curved lamella shape. The particle size distribution was illustrated in Figure 4. There was no obvious stacking of O-MoS_2_ lamellae, which is in good accordance with the XRD analysis. The O-MoS_2_ exhibited a monolayer structure with lamellae length ranging from 5 to 14.0 nm. By comparison, W-MoS_2_ possessed a longer lamellae length of 8–18 nm with a stacking number of 5–13. Since the nanocatalyst with a smaller grain size typically facilitates the exposition of edge sites, better catalytic activity could be expected of O-MoS_2_ [44]. Mo_2_C samples exhibited various dispersed nanocrystalline particles with the average size of 7–19 nm. The difference between the mean size obtained from XRD and that from TEM could possibly be ascribed to the irregular shape of MoS_2_ lamellae or Mo_2_C particle. The agglomeration could be observed in some Mo_2_C nanoparticles. The *d* lattice spacing of 0.237 nm was found in Mo_2_C, assigned to the hexagonal β-Mo_2_C (002) planes, which further confirms the formation of molybdenum carbide.

The chemical states for these Mo-based nanoparticles were examined by XPS. All the binding energy was calibrated using the C 1s photoelectron peak at 284.8 eV as a reference. Figure 4 presents the high-resolution XPS spectra of Mo 3d, S 2p for the O-MoS_2_ and W-MoS_2_, and Mo 3d, C 1s for the Mo_2_C. In the XPS spectra of the Mo 3d and S 2s region, the two major peaks located at 229.17 and 232.32 eV correspond to Mo^4+^ 3d_5/2_ and Mo^4+^ 3d_3/2_ of the MoS_2_, the major peaks at 162.0 and 163.17 eV are attributed to S 2p_3/2_ and S 2p_1/2_ of the MoS_2_, respectively [45,46]. In addition to the Mo 3d signals, a peak at 226.3 eV belonging to the S 2s orbital was observed. Obviously, a small peak at 235.8 eV can be ascribed to the Mo^6+^ of MoO_3_, which was ascribed to the inevitable surface oxidation of MoS_2_ when exposed in air. The Mo 3d high-resolution element spectrum of Mo_2_C was deconvoluted into six peaks, in which two peaks at 228.8 eV (3d_5/2_) and 231.9 eV (3d_3/2_) can be assigned to Mo^2+^ in Mo_2_C, two peaks at 230.1eV and 233.2 eV denote Mo^4+^ in Mo_2_C or MoO_2_ and two peaks at 232.8 eV and 235.9 eV belong to Mo^6+^ in MoO_3_ [47]. The appearance of MoO_2_ and MoO_3_ is usually caused by the use of inert gas atmosphere with 1% oxygen in the passivation process or the oxidation of carbides exposed to air, which is consistent with previous literature [48,49]. The peaks of 284.4 and 285.7 eV in the spectra of C 1s belong to C-Mo and C-C bonds in Mo_2_C. The atomic ratio of C/Mo was calculated to be 1:2.08 based on the XPS data, indicating the dominance of Mo_2_C in the as-synthesized catalyst.

**Figure 5:**
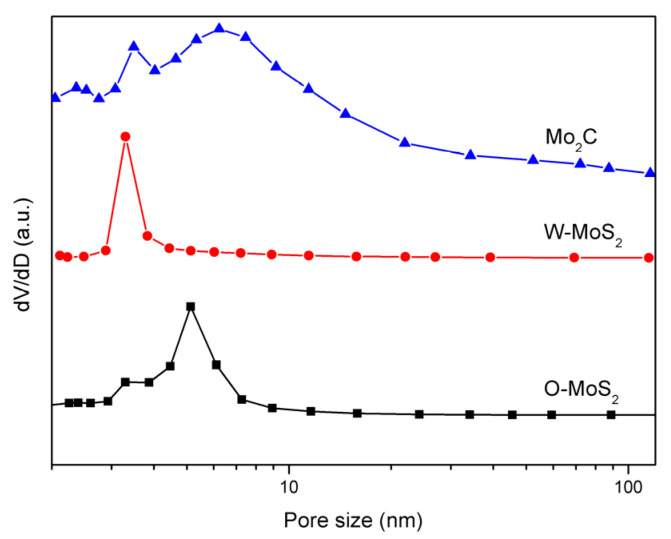
Pore-size distribution curves of catalyst samples.

The BET surface areas of the W-MoS_2_, O-MoS_2_ and Mo_2_C were determined to be 51.6, 324.6 and 42 m^2^/g, respectively. The extremely high surface area of O-MoS_2_ was much greater than that of W-MoS_2_ and could be one of the possible reasons for its higher catalytic activity. Even though Mo_2_C presented a lower surface area than MoS_2_, it could still display excellent catalytic activity because of its Pt-like nature. The BJH pore size distribution curves of MoS_2_ and Mo_2_C are presented in Figure 5. The pore-size distributions revealed that the W-MoS_2_ and O-MoS_2_ had only a narrow peak at around 3.2 nm and 9 nm, respectively. Conversely, the Mo_2_C catalyst showed a narrow pore-size distribution peak at approximately 3.4 nm, and another larger pore distribution broader than 5 nm. Since larger mesopores are much more important for mass transfer resistance for reacting molecules, such a combination on the hierarchical structures is potentially ideal for the catalytic reaction. The hierarchical structures prevent the entry of polycyclic aromatic hydrocarbons, but allow the entry of olefinic substituents. This special pore size distribution could be beneficial for improving the selectivity in the hydrogenation reaction.

### 3.2. Hydrogenation Activity Measurement in Slurry Oil Surrogate

**Figure 6:**
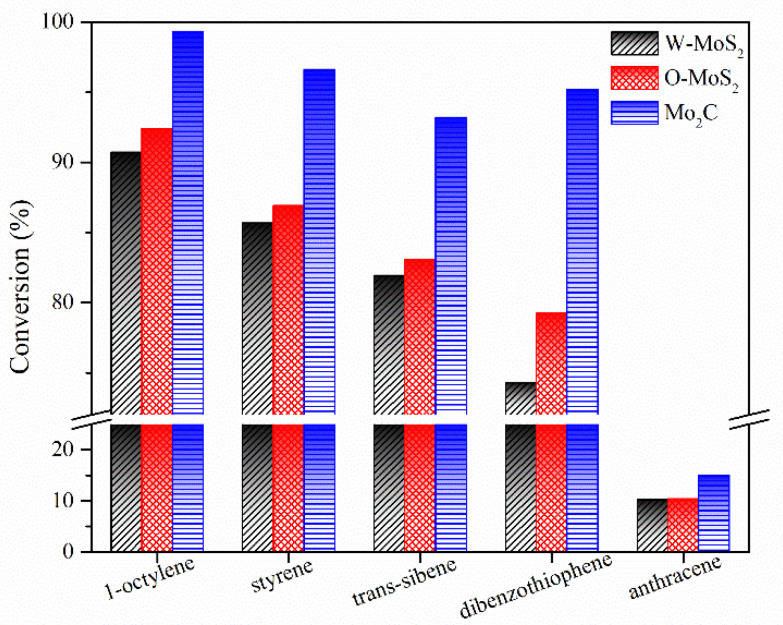
Conversion of model compounds on various catalysts.

**Figure 7:**
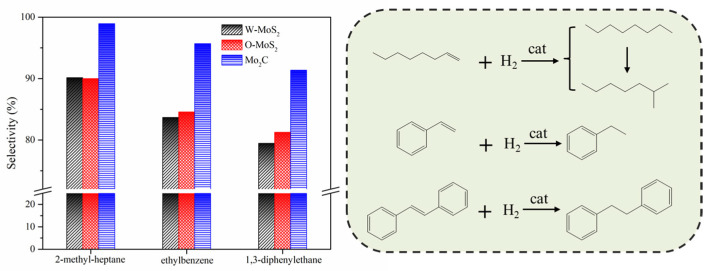
Main product selectivity and hydrogenation pathway of olefins on various catalysts.

The hydrogenation treatments of the slurry oil surrogate were carried out at 380 °C with MoS_2_ and Mo_2_C nanoparticles. The typical GC-MS results of the products are shown in Figure S1 as supporting information. It can be seen that 1-methylnaphthalene was simultaneously hydrogenated and thermally cracked during the hydrotreating process, and the chromatographic peaks of these derived products were relatively extensive because of the extremely high amount of 1-methylnaphthalene. However, it did not affect the following discussion about the catalytic activity of Mo-based catalysts for the hydrogenation of olefins and sulfur compounds in the present work. The conversions for each molecule contained in the slurry oil surrogate are shown in Figure 6. Over 80% of the olefins were removed by hydrotreatment with the three Mo-based nanoparticles. The conjugated olefins are more difficult to hydrogenate than monoolefins due to the higher aromaticity and larger steric hindrance [7]. Because of the good dispersion and smaller particles, proved by HRTEM, the O-MoS_2_ exhibited better activity for olefin removal than W-MoS_2_, although the amount of W-MoS_2_ was greater [24]. Further enhanced catalytic activity was found for Mo_2_C, even trans-stilbene had an increased conversion of 92%. The selectivity for hydrogenated products during olefin hydrotreatment is illustrated in Figure 7. It demonstrates that the excellent catalytic performance of Mo_2_C for olefin removal mainly originates from its activity for hydrogenation. For the three olefins, the 1,3-diphenylethane, ethylbenzene and 2-methyl-heptane selectivity can be up to 93.2%, 96.6% and 99.3%, respectively.

**Figure 8:**
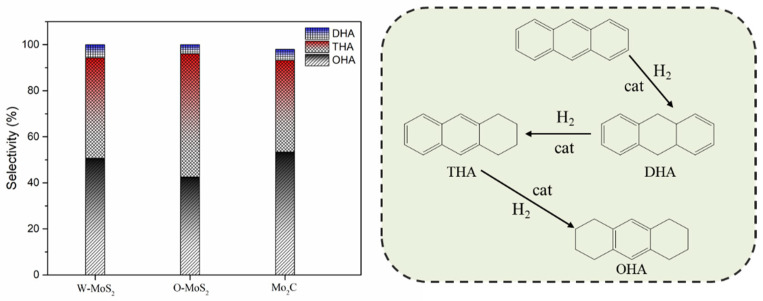
Product selectivity and hydrogenation pathway of anthracene. DHA, THA and OHA refer to 9,10-dihydroanthracene, 1,2,3,4-tetrahydroanthracene, and 1,2,3,4,5,6,7,8-octahydroanthracene.

The activity improvement of Mo_2_C could also be observed for catalyzing sulfur removal and tended to be much stronger. This was consistent with previous reports [40]. The conversion of dibenzothiophene rose from 65% with W-MoS_2_ to 95% with Mo_2_C. Anthracene presented the lowest conversion compared with olefins and dibenzothiophene, and there was no great difference for the three catalysts, indicating Mo_2_C displayed a good selectivity for olefins and dibenzothiophene conversion. Figure 8 shows the product selectivity for anthracene conversion with these three catalysts. It was found that Mo_2_C facilitated the formation of 9,10-dihydroanthracene, suggesting that Mo_2_C could limit the anthracene hydrogenation to a mild hydrogenation step and protect it from being deeply hydrogenated. According to the literature [1], the production of a small portion of hydroaromatics could serve as hydrogen donors during the carbonization of FCC slurry oil and benefit the good morphology of coke formation.

### 3.3. Hydrogenation Activity Evaluation in FCC Slurry Oil

The catalytic performance of these Mo-based catalysts on the hydrotreating of FCC slurry oil were further evaluated. The elemental analysis and molecular weight of FCC slurry oil before and after the hydrotreating process with different catalysts are compared in Table 2. The H/C ratio presented only a slight increase after hydrotreatment and the molecular weight did not change much. It indicates that FCC slurry oil has been subjected to a mild hydrotreating process, which is necessary to preserve the main native polyaromatics components. The major change of FCC slurry oil in the elemental analysis lies in the sulfur content which decreased from 0.41% to approximately 0.22%. A portion of nitrogen was also removed during this process, even though the removal efficiency was lower because of the higher bonding energy of C-N than C-S.

**Figure 9:**
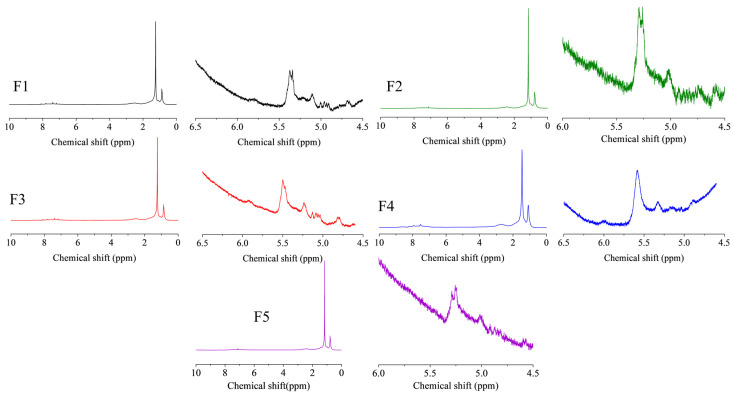
The ^1^H-NMR spectra of FCC slurry oil before and after hydrotreatment. The feed FCC slurry oil is designated as F1; FCC after reaction with non-catalyst is designated as F2. FCC after reaction with A-MoS_2_ is designated as F3; FCC after reaction with M-MoS_2_ is designated as F4; FCC after reaction with Mo_2_C is designated as F5.

Figure 9 shows the ^1^H-NMR spectra of FCC slurry oil before and after hydrogenation, and the calculated hydrogen distribution is listed in Table 3. The hydrogen type classification was referenced from previous literature [7,50]. A slight decrease of the aromatic hydrogen content (H*_car_*) was found in this study, suggesting the mild hydrogenation of aromatics [51]. Consequently, the content of naphthenic hydrogen (H_cβ_ and H_c__α_) has been raised. As aforementioned, the generation of these naphtheno-aromatics is desirable since they could benefit the hydrogen transfer behavior during the carbonization of SLO [1]. Furthermore, the aliphatic hydrogen of methyl or methylene groups in the α- and *γ*-position to an aromatic ring was observed to be increased while that in the β-position to an aromatic ring was reduced. It suggests that the thermal cracking reactions of aliphatic carbon chains have occurred, which could be due to the cracking function of these nanoparticles. More importantly, the olefin hydrogen content (H*_o_*) was significantly decreased, indicating the excellent catalytic activity for olefin removal.

To further evaluate the transformation of the FCC slurry oil, the average molecular structural parameters were then calculated based on the modified Brown-Ladner method, the main calculation equations are provided in Table S2 as the supporting information, with the results presented in Table 4. Apparently, even though *R_A_* showed a slight decrease because of the hydrogenation reactions, resulting in an increase of *R_N_*, there was no significant difference for the *f_A_*, *f_N_* and *f_P_* of hydrotreated oil, proving that the aromatic moiety of SLO was protected from overhydrogenation.

**Figure 10:**
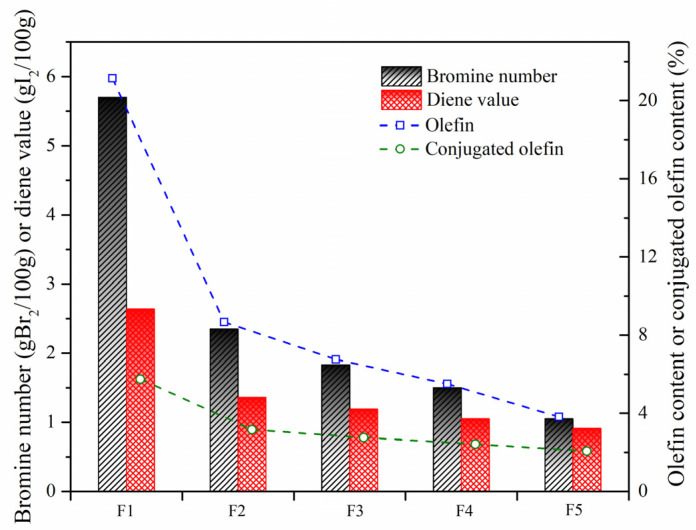
Bromine numbers and diene values of SLO before and after hydrogenation. The feed FCC slurry oil is designated as F1; FCC after reaction with non-catalyst is designated as F2. FCC after reaction with A-MoS_2_ is designated as F3; FCC after reaction with M-MoS_2_ is designated as F4; FCC after reaction with Mo_2_C is designated as F5.

In order to further investigate olefin removal with the aid of these nanoparticles, the bromine number and diene value were measured. As shown in Figure 10, the catalytic activity of W-MoS_2_, O-MoS_2_ and Mo_2_C to remove olefins was improved successively, which was consistent with the observation with the model compound. With Mo_2_C, the olefins had significantly decreased from 5.7 to 1.05 gBr_2_/100 g, and the conjugated olefins were reduced from 2.64 to 0.91 gI_2_/100 g. Based on the formulation as Equations (1) and (2), the content of olefins can be calculated.

w_o_ = V_Br_ × M/(2 × 79.9) (1)

w_co_ = V_Di_ × M/253.8 (2)

where w_o_ and w_co_ are the content of olefins and conjugated olefins, respectively. V_Br_ and V_Di_ refer to the measured bromine value and diene value, respectively. M is the molecular weight of the sample. The results showed that the feed SLO contained about 21.14% olefins. Among the olefins, the proportion of conjugated olefins could be 27%. As systematically stated by Jiao et al. [10], these olefinic molecules in considerable amounts were extremely reactive in generating free radicals, which would aggravate the internal thermal reactions governing the stability of SLO and affecting the quality of the carbonaceous materials prepared. After hydrotreatment with O-MoS_2_ and Mo_2_C, only 5.5% and 3.8% of olefins were left in the SLO.

**Figure 11:**
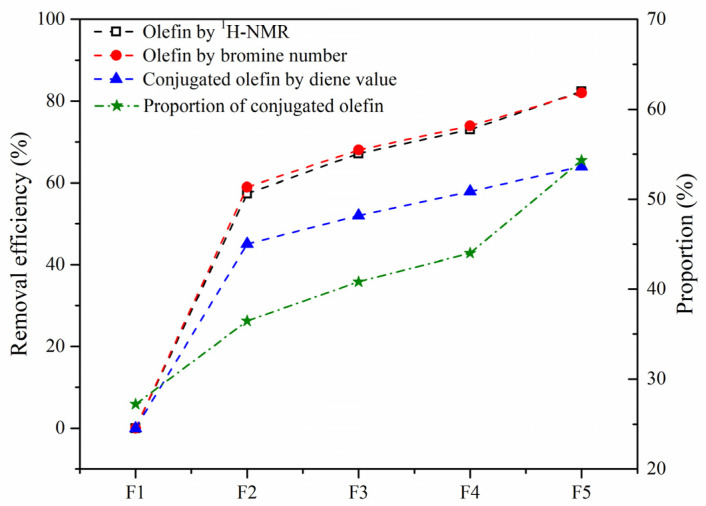
Removal efficiency of olefins in the SLO. The feed FCC slurry oil is designated as F1; FCC after reaction with non-catalyst is designated as F2. FCC after reaction with A-MoS_2_ is designated as F3; FCC after reaction with M-MoS_2_ is designated as F4; FCC after reaction with Mo_2_C is designated as F5.

The removal efficiency of olefins by hydrotreating based on the bromine value and H*_o_* content and that of the conjugated olefins based on diene value are compared in Figure 11. The good agreement of the results by the two indicators suggests that ^1^H-NMR is an effective and convenient technique for analyzing the olefin content. The conversion of conjugated olefins was found to be much lower than that of the total olefins, leading to the proportion of conjugated olefins to olefins gradually increasing. Compared with MoS_2_, Mo_2_C showed the highest catalytic activity for olefin removal, and the conversion of olefins and conjugated olefins could be up to 82% and 64%, respectively.

**Figure 12:**
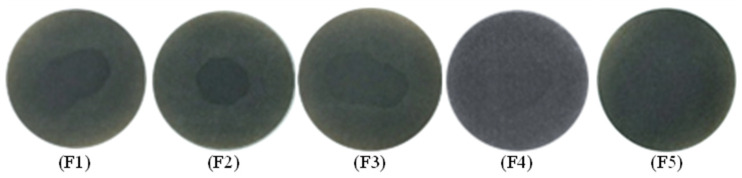
Spot experiment of FCC slurry oil before and after hydrogenation on various catalysts. The feed FCC slurry oil is designated as F1; FCC after reaction with non-catalyst is designated as F2. FCC after reaction with A-MoS_2_ is designated as F3; FCC after reaction with M-MoS_2_ is designated as F4; FCC after reaction with Mo_2_C is designated as F5.

To describe the thermal stability of slurry oil, the spot experiment was carried out, with the results displayed in Figure 12. F1 shows obvious dark rings inside the spot indicating the poor stability of the feed oil, which can be mainly attributed to the existence of abundant active olefins. Therefore, the olefinic bonds must be hydrogenated to be removed. Mild hydrogenation treatment alone or with W-MoS_2_ were not so effective in improving the stability of SLO. In contrast, when O-MoS_2_ catalysts were added into the reaction system, the stability of the slurry oil was obviously improved, which was attributed to the good dispersion and strong catalytic hydrogenation activity of MoS_2_. Additionally, the spot experiment of slurry oil under Mo_2_C showed that almost no dark rings appeared, implying high hydrogenation activity of the Mo_2_C catalyst which effectively enhanced the thermal stability of SLO.

## 4. Conclusions

Olefins and sulfur are the major impurities widely distributed in FCC slurry oil (SLO) which seriously deteriorate the preparation and quality of mesophase pitch or needle coke. The development of a hydrotreatment for SLO to remove olefins and sulfur selectively becomes imperative. This work presents the potentiality of dispersed Mo_2_C and MoS_2_ nanoparticles as selective hydrotreating catalysts of SLO. Mo_2_C was synthesized by the carbonization of citric acid, ammonium molybdate and KCl mixtures while MoS_2_ was prepared from the decomposition of precursors. These catalysts were characterized by XRD, HRTEM, XPS, BJH, and applied to the hydrotreatment of the SLO surrogate with defined components and the real SLO. The results showed that hydrotreating of the SLO surrogate with a very small amount of Mo-based nanoparticles could selectively remove olefins and sulfur without overhydrogenation of polyaromatics. Mo_2_C exhibited much better activity than MoS_2_, with 95% of olefins and dibenzothiophene in the surrogate removed. For the real SLO study, approximately 50% sulfur and 82% olefins were removed with Mo_2_C, and the stability was significantly improved. The calculated structural parameters were changed subtly, proving that the aromatic macromolecules were not affected. Additionally, it was found that the removal efficiency of olefins calculated by bromine number was in good agreement with that obtained by ^1^H-NMR, suggesting ^1^H-NMR is an effective and convenient technique to analyze the olefin content for SLO.

## Figures and Tables

**Table 1:** Properties of the real SLO feedstock.

Items	Value
Density ρ_20_/(g·cm^−3^)	0.9773
Kinematic viscosity η_80_/(mm^2^·s^−1^)	98.60
Kinematic viscosity η_100_/(mm^2^·s^−1^)	50.10
Solid content wt/%	0.26
Carbon residue wt/%	10.76
Ash wt/%	0.16
Ni (µg·g^−1^)	67.84
V (µg·g^−1^)	<3.0
Saturates (wt%)	34.64
Aromatics (wt%)	45.23
Colloid (wt%)	19.49
C7-asphaltenes (wt%)	0.64
Density ρ_20_/(g·cm^−3^)	0.9773
Kinematic viscosity η_80_/(mm^2^·s^−1^)	98.60

**Table 2:** Distribution of C/H/S/N elements in FCC slurry oil before and after hydrotreatment.

Samples	F1	F2	F3	F4	F5
C/%	87.79	87.80	87.81	87.84	87.86
H/%	11.25	11.29	11.33	11.39	11.41
S/%	0.41	0.32	0.26	0.24	0.22
N/%	0.31	0.29	0.27	0.25	0.23
H/C	1.54	1.54	1.55	1.56	1.56
molecular weight	593	591	590	585	580

Note: the feed FCC slurry oil is designated as F1; FCC after reaction with non-catalyst is designated as F2. FCC after reaction with A-MoS_2_ is designated as F3; FCC after reaction with M-MoS_2_ is designated as F4; FCC after reaction with Mo_2_C is designated as F5.

**Table 3:** Hydrogen type distribution in the ^1^H-NMR spectra of FCC slurry oil before and after hydrotreatment.

Chemical Shift (ppm)	Hydrogen Type	Symbol	F1	F2	F3	F4	F5
0.5–1.0	Terminal methyl hydrogens of paraffins or of alkyl side chains three or more positions from an aromatic ring.	H_γ_	22.58	23.10	23.29	23.90	23.95
1.0–1.4	Interior hydrogens of paraffins. Methyl hydrogens two positions from an aromatic ring. Non-cyclic methylene or methylidyne hydrogens two or more positions from an aromatic ring.	H_β_	44.16	43.98	43.61	42.40	41.87
1.4–2.0	Naphthenic hydrogens. Naphthenic hydrogens two positions from the aromatic ring of naphtheno-aromatics.	H_cβ_	7.64	7.91	8.14	8.45	8.64
2.0–2.5	Methyl or non-cyclic methylene or methylidyne hydrogen adjacent to aromatic ring.	H_α_	5.87	6.15	6.27	6.64	6.82
2.5–4.5	Hydrogen on naphthenic carbon adjacent to fused aromatic ring of naphtheno-aromatic.	H_cα_	6.36	6.82	6.96	7.28	7.54
4.5–6.5	Hydrogens in olefin.	H_o_	2.04	0.87	0.67	0.55	0.36
6.5–9.0	Hydrogens in aromatic ring.	H_car_	11.35	11.17	11.06	10.78	10.82

Note: the feed FCC slurry oil is designated as F1; FCC after reaction with non-catalyst is designated as F2. FCC after reaction with A-MoS_2_ is designated as F3; FCC after reaction with M-MoS_2_ is designated as F4; FCC after reaction with Mo_2_C is designated as F5.

**Table 4:** The average molecular structural parameters of FCC slurry oil before and after hydrotreatment.

Structural Parameters	F1	F2	F3	F4	F5
*f_A_*	0.32	0.32	0.31	0.31	0.31
*f_N_*	0.10	0.11	0.11	0.11	0.11
*f_P_*	0.58	0.58	0.58	0.59	0.59
*R_A_*	2.96	2.90	2.86	2.77	2.73
*R_N_*	1.10	1.14	1.14	1.16	1.14
*R_T_*	4.06	4.04	4.00	3.93	3.87

Note: *f_A_* is the aromaticity, *f_N_* is the naphthenic carbon ratio, *f_P_* is the paraffinic carbon ratio, *R_A_* is the aromatic ring number, *R_N_* is the naphthenic ring number, *R_T_* is the total carbon number. The feed FCC slurry oil is designated as F1; FCC after reaction with non-catalyst is designated as F2. FCC after reaction with A-MoS_2_ is designated as F3; FCC after reaction with M-MoS_2_ is designated as F4; FCC after reaction with Mo_2_C is designated as F5.

## Data Availability

The data presented in this study are available on request from the corresponding author.

## Supplementary Materials

The following are available online at https://www.mdpi.com/article/10.3390/nano11102721/s1, Figure S1: The typical chromatogram of products from hydrotreating of SLO surrogate, Table S1: Specification of the chemicals used in this work, Table S2: Equations for the modified Brown-Ladner method.
