# Supplementary material for: Molybdenum Carbide and Sulfide Nanoparticles as Selective Hydrotreating Catalysts for FCC Slurry Oil to Remove Olefins and Sulfur
Source: Nanomaterials (Basel). 2021 Oct 15;11(10):2721. doi: 10.3390/nano11102721 (PMC8540075; doi:10.3390/nano11102721)
Supplement: Supplementary file 1 [file nanomaterials-11-02721-s001.zip › nanomaterials-1404832-supplementary.pdf]

Supplementary Materials

Molybdenum Carbide and Sulfide Nanoparticles as Selective Hydrotreating Catalysts for FCC Slurry Oil to Remove Olefins and Sulfur

He Liu, Zhipeng Qiu *, Huihui Pan, Aijun Guo *, Shouhui Jiao, Feng Wang, Kun Chen and Zongxian Wang

State Key Laboratory of Heavy Oil Processing, College of Chemical Engineering, China University of Petroleum (East China), 66 Changjiang West Road, Huangdao District, Qingdao 266580, Shandong, China; liuhe@upc.edu.cn (H.L.); panhuihui_upc@163.com (H.P.); b18030013@s.upc.edu.cn (S.J.); wfzgsydx123@163.com (F.W.); chenkun@upc.edu.cn (K.C.); james_w3112@163.com (Z.W.)

* Correspondence: z19030042@s.upc.edu.cn (Z.Q.); ajguo@upc.edu.cn (A.G.)

Table S1. Specification of the chemicals used in this work

chemical name	CAS no.	purity provided by the supplier	source
		mass fraction	
ammonium molybdate tetrahydrate	12054-85-2	99.7%	Sinopharm Chemical Reagent Co., Ltd, China
citric acid monohydrate	5949-29-1	99.7%	Sinopharm Chemical Reagent Co., Ltd, China
potassium chloride	7447-40-7	99.7%	Sinopharm Chemical Reagent Co., Ltd, China
ethylene glycol	107-21-1	99.7%	Sinopharm Chemical Reagent Co., Ltd, China
nitric acid	7697-37-2	99.7%	Sinopharm Chemical Reagent Co., Ltd, China
molybdenum dialkyl dithiocarbamate	71342-87-7	99.7%	Sinopharm Chemical Reagent Co., Ltd, China
1-methylnaphthalene	90-12-0	99.7%	Sinopharm Chemical Reagent Co., Ltd, China
sulfur	7704-34-9	99.7%	Sinopharm Chemical Reagent Co., Ltd, China
styrene	100-42-5	99.7%	Sinopharm Chemical Reagent Co., Ltd, China

trans-stilbene	103-30-0	98%	Saen Chemical Technology Co., Ltd, China
1-octene	111-66-0	99%	Sinopharm Chemical Reagent Co., Ltd, China
dibenzothiophene	95-15-8	98%	Shanghai Macklin Biochemical Co., Ltd, China
anthracene	120-12-7	99.5%	Shanghai Macklin Biochemical Co., Ltd, China

Table S2. Equations for the modified Brown-Ladner method

Symbol	Structural parameter for an unit	Equation
H_T	total hydrogen (olefinic hydrogen excluded)	$H_{\mathrm{T}}=H_{\alpha}+H_{c\alpha}+H_{\beta}+H_{c\beta}+H_{\gamma}$
f_A	aromaticity	$f_{\mathrm{A}}=\frac{C/H-(H_{\alpha}+H_{\beta}+H_{\gamma})/2H_{\mathrm{T}}}{C/H}$
H_{AU}/C_A	condensation degree of aromatic rings	$H_{\mathrm{AU}}/C_{\mathrm{A}}=\frac{H_{\mathrm{A}}/H_{\mathrm{T}}+(H_{\alpha}+H_{c\alpha})/2H_{\mathrm{T}}}{C/H-(H_{\alpha}+H_{c\alpha}+H_{\beta}+H_{c\beta}+H_{\gamma})/2H_{\mathrm{T}}}$
C_A	aromatic carbon number	$C_{\mathrm{A}}=\frac{3}{(H_{\mathrm{AU}}/C_{\mathrm{A}})-0.5}$ [a] or $C_{\mathrm{A}}=\left(\frac{2.503}{(H_{\mathrm{AU}}/C_{\mathrm{A}})}\right)^2$ [b]
C_T	total carbon number	$C_{\mathrm{T}}=\frac{C_{\mathrm{A}}}{f_{\mathrm{A}}}$
R_A	aromatic ring number	$R_{\mathrm{A}}=(C_{\mathrm{A}}-2)/4$ [a] or $R_{\mathrm{A}}=(C_{\mathrm{A}}-4)/3$ [b]
R_N	naphthenic ring number	$R_{\mathrm{N}}=C_{\mathrm{T}}+1-H_{\mathrm{T}}/2-C_{\mathrm{A}}/2-R_{\mathrm{A}}$
C_N	naphthenic carbon number	$C_{\mathrm{N}}=4R_{\mathrm{N}}$ [a] or $C_{\mathrm{N}}=3R_{\mathrm{N}}$ [b]
f_P	paraffinic carbon ratio	$f_{\mathrm{P}}=C_{\mathrm{P}}/C_{\mathrm{T}}=(C_{\mathrm{T}}-C_{\mathrm{T}}f_{\mathrm{A}}-C_{\mathrm{N}})/C_{\mathrm{T}}$
f_N	naphthenic carbon ratio	$f_{\mathrm{N}}=C_{\mathrm{N}}/C_{\mathrm{T}}$

Note: [a] For cata-condensed aromatic system ((H_{AU}/C_A)≥0.5). [b] For peri-condensed aromatic system ((H_{AU}/C_A)<0.5).

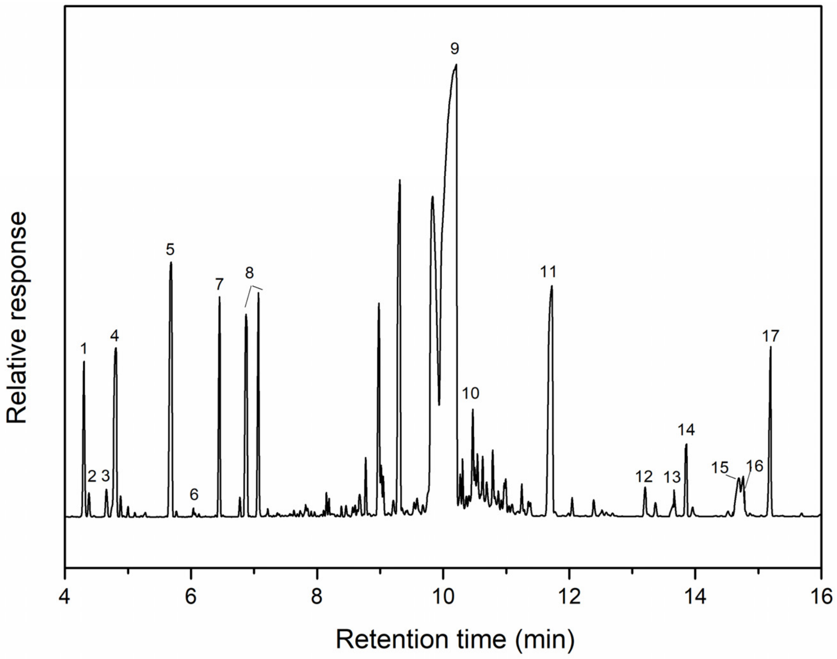

Figure S1. The typical chromatogram of products from hydrotreating of SLO surrogate. Numbers: 1=solvent (i.e. toluene), 2=2-methylheptane, 3=1-octylene, 4=3-ethylhexane, 5=ethylbenzene, 6=styrene, 7=1-ethylmethylbenzene, 8=1-ethyl-2-methyl-benzene, 9=1-methylnaphthalene, 10=biphenyl, 11=bibenzyl, 12=trans-sibene, 13=9,10-dihydroanthracene, 14=1,2,3,4,5,6,7,8-octahydroanthracene, 15=1,2,3,4-tetrahydroanthracene, 16=dibenzothiophene, 17=anthracene.
